# Politeness and Compassion Differentially Predict Adherence to Fairness Norms and Interventions to Norm Violations in Economic Games

**DOI:** 10.1038/s41598-017-02952-1

**Published:** 2017-06-13

**Authors:** Kun Zhao, Eamonn Ferguson, Luke D. Smillie

**Affiliations:** 1 The University of Melbourne, Melbourne School of Psychological Sciences, Melbourne, 3010 Australia; 2 University of Nottingham, School of Psychology, Nottingham, NG7 2RD United Kingdom

## Abstract

Adherence to norms and interventions to norm violations are two important forms of social behaviour modelled in economic games. While both appear to serve a prosocial function, they may represent separate mechanisms corresponding with distinct emotional and psychological antecedents, and thus may be predicted by different personality traits. In this study, we compared adherence to fairness norms in the dictator game with responses to violations of the same norms in third-party punishment and recompensation games with respect to prosocial traits from the Big Five and HEXACO models of personality. The results revealed a pattern of differential relations between prosocial traits and game behaviours. While norm adherence in the dictator game was driven by traits reflecting good manners and non-aggression (the politeness aspect of Big Five agreeableness and HEXACO honesty-humility), third-party recompensation of victims—and to a lesser extent, punishment of offenders—was uniquely driven by traits reflecting emotional concern for others (the compassion aspect of Big Five agreeableness). These findings demonstrate the discriminant validity between similar prosocial constructs and highlight the different prosocial motivations underlying economic game behaviours.

## Introduction

Economic games are models of social interactions involving fairness, cooperation, trust, and punishment^1^. Recently, diverse lines of research have begun to converge on the broad classes of social behaviours captured in these games. Specifically, the discovery that fair and cooperative behaviours are correlated across several economic games and over time has pointed to a broad prosocial tendency^2–4^, dubbed the *cooperative phenotype* ^4^. Another social behaviour modelled in economic games involves interventions to violations of these norms, which can be expressed through punishment of offenders and assistance to victims. Indeed, norm-enforcing punishment behaviours in various games have been found to cluster together and form a factor orthogonal to the cooperative phenotype^4^.

A potential source of these apparent behavioural phenotypes are broad personality traits capturing consistent and enduring patterns in thoughts, feelings, and behaviours. Recent developments in the integration of personality psychology and economics provide a better understanding of how behavioural heterogeneity in economic games can be explained by personality traits^5, 6^. Personality traits can be defined as “probabilistic descriptions of relatively stable patterns of emotion, motivation, cognition, and behavior, in response to classes of stimuli that have been present in human cultures over evolutionary time” (p. 35)^7^. These are classified according to major patterns of covariation within hierarchical models, so that narrower traits (i.e., facets) are grouped together to form broader traits at the intermediate (i.e., aspects) and higher (i.e., domain) levels. Prosocial domains in these models refer to an array of related traits which confer benefits on other individuals, such as the tendencies to be kind, forgiving, helpful, trusting, fair, and sincere. These are organised differently in two major models of personality, the Big Five^8–10^ and the HEXACO (Honesty-Humility, Emotionality, eXtraversion, Agreeableness, Conscientiousness, Openness to Experience)^11^.

In the Big Five, prosocial traits primarily fall within the broad domain of agreeableness, which describes a general tendency to be altruistic^12^. Agreeableness subsumes multiple prosocial tendencies (e.g., warmth, sympathy, modesty), which can be classed into two trait “aspects”: compassion—the tendency to be emotionally concerned about others (e.g., “Sympathise with others’ feelings”), and politeness—the tendency to be respectful of others and to suppress aggressive, norm-violating impulses (e.g., “Take advantage of others”, reversed)^13^. While the two are related, they are each separately linked to individual differences in political ideology and moral values, and are thought to reflect different biological substrates^14, 15^.

The HEXACO is an alternative six-factor model of personality, in which prosocial constructs are separated into two major domains corresponding to two complementary aspects of reciprocal altruism: honesty-humility and agreeableness^16, 17^. One notable feature of the HEXACO is its sixth domain, honesty-humility, which reflects the tendency to be fair-minded, sincere, modest, and non-greedy (e.g., “I wouldn’t pretend to like someone just to get that person to do favours for me”). Honesty-Humility represents individual differences in *active cooperation*, where one behaves cooperatively with others despite the temptation to exploit them without repercussion^16, 18, 19^. While it captures trait variance beyond that of the Big Five^20^, it is conceptually similar to the *politeness* aspect of Big Five agreeableness, as both traits are marked by low aggression and exploitativeness^13, 21^. In addition, HEXACO agreeableness reflects the tendency to be patient, forgiving, and tolerant (e.g., “I rarely hold a grudge, even against people who have badly wronged me”). Thus, it is not interchangeable with Big Five agreeableness, which is a broader prosocial domain. Unlike honesty-humility, it represents individual differences in *reactive cooperation*, where one behaves cooperatively with others despite their transgressions^16, 18, 19^.

## Personality Traits Associated with Adherence to Fairness and Cooperation Norms

Recent research has focused on the role of prosocial traits from the Big Five and HEXACO models in various forms of active cooperation in economic games. These studies have consistently linked the politeness aspect of Big Five agreeableness and HEXACO honesty-humility to the fair division of resources in the dictator game^6, 22–24^ (a simple task in which one player divides an endowment with a recipient), and the latter with greater cooperation in the prisoner’s dilemma^25^, contributions in public goods games^26^, and returns in the trust game^27^. This ties in with evidence of a domain-general and temporally-stable prosocial tendency—the cooperative phenotype—which comprises fair and cooperative behaviours correlated across these same economic games^3, 4^. Therefore, one possibility is that self-reported politeness/honesty-humility from robust taxonomies of personality manifest behaviourally as the cooperative phenotype in economic games.

## Personality Traits Associated with Interventions to Norm Violations

Interventions to redress norm violations are a second type of social behaviour that features prominently in economic games^28, 29^, even when interactions are anonymous and there is little to gain from direct reciprocity or reputation^30^. Punishment, in particular, is crucial for maintaining social order, and experiments of public goods games show that cooperation breaks down quickly in the absence of punishment of norm violations such as free-riding^28, 29^. Responses to norm violations can be studied using the *third-party punishment game*, in which an uninvolved and impartial outside party bears a cost to punish an individual who has exploited a powerless victim^30^. This has revealed a substantial proportion of individuals—up to 60%—willing to spend their own money to punish norm violators, even when they were not directly harmed and nor would reap any benefits^30^. Negative moral emotions play a key part in this process, with anger and judgments of unfairness associated with punishment^28, 30–32^.

Costly third-party punishment can, therefore, be interpreted as an altruistic act oriented towards the preservation of societal or ethical norms. Unlike its *second-party* counterpart (where an individual is both the victim and the punisher), third-party punishment uncouples prosocial motives from spiteful or status-defending mechanisms. Accordingly, third-party punishers are also seen as more trustworthy^33^ and are more often rewarded by others^34^.

While punishment is the standard response to norm violations in economic games, multiple routes to justice restoration exist in the real world. These can generally be directed toward the perpetrator or victim^35^. The latter is captured by the third-party *recompensation* game, in which a participant can incur a cost to recompense the victim^36, 37^. Punishment and recompensation are thus alternative and contrasting approaches in the restoration of justice—even though both may serve the perpetrator or victim their just deserts, only the former can deter future acts of norm violation and only the latter can alleviate a victim’s suffering^35, 36^.

Although interventions to norm violations are widespread, they are also subject to inter-individual variation which may reflect different prosocial motivations arising from personality traits. For example, research has examined the role of traits concerning empathy in third-party recompensation games. Here, individuals high on empathic concern, or the tendency to feel sympathy and concern for unfortunate others^38^, transferred greater amounts of their own money to victims of unfair distributions of wealth^36, 37^. Similarly, when faced with the decision to punish, recompense, or do neither as the third party, individuals high on empathic concern recompensed more frequently and were faster in their decisions^39^. The research on personality traits underpinning punishment is less clear and at times is at odds with the economic games literature. For example, some studies have reported a *negative* trend between empathic concern and the amount of money spent on punishment^36, 39^, which may be driven by antisocial punishers who both spent money on punishment and withheld resources selfishly in the dictator game^37^.

## The Current Study

**Figure 1:**
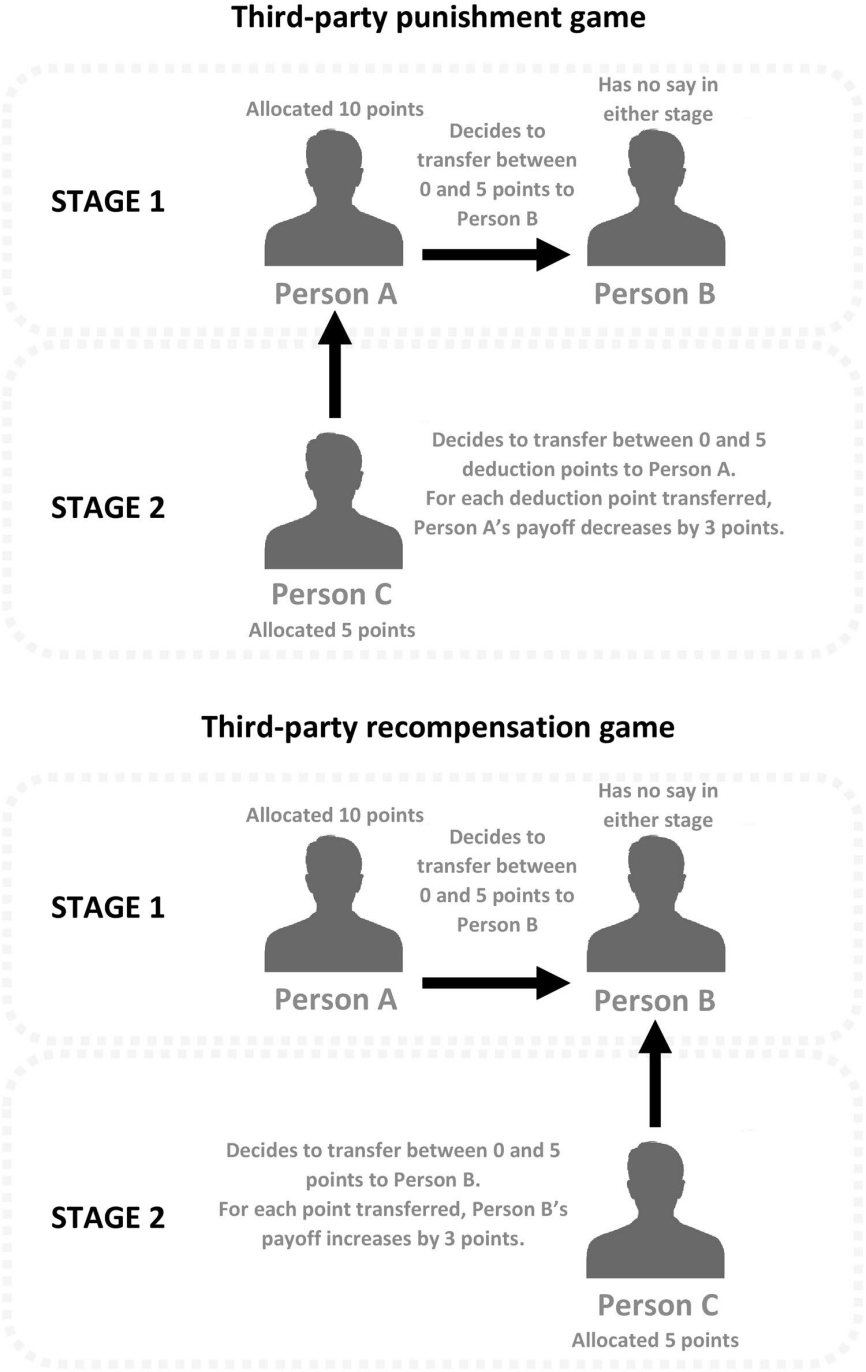
Study designs of the third-party games.

Bringing together these different lines of research, our aim was to examine the sources of individual differences in two types of economic game behaviours: adherence to fairness norms in the dictator game and interventions to violations of these same norms in third-party games (i.e., the third-party punishment, 3PP, and third-party recompensation, 3PR, games, depicted in Fig. 1). We mapped these to the prosocial domains from two major models of personality: the politeness and compassion aspects of Big Five agreeableness, and HEXACO honesty-humility and agreeableness. We included comprehensive personality measures spanning the prosocial domain in order to identify unique trait effects and dissociations between similar prosocial constructs sharing common characteristics of general benevolence. In addition, our study design allowed us to address some limitations of previous research, by (1) disentangling multiple motives for punishment by examining third-party responses in conjunction with allocations of wealth in the dictator game, (2) drawing from larger and adequately-powered samples, and (3) using multiple comprehension questions and attention checks for data quality. This last consideration is important given recent evidence of high proportions of participants who had difficulty understanding related economic games^40^.

In line with the previous literature, we expected to replicate the link between adherence to fairness norms and the conceptually-similar traits of politeness and honesty-humility. We further predicted that interventions to norm violations would be related to compassion, given its conceptual similarity to empathic concern and its core feature of emotional concern for others, which would increase the focus on the victim and moral outrage towards the offender. Lastly, we were interested in the role of HEXACO agreeableness given its theoretical importance for reactive cooperation with others despite their transgressions^16^. While HEXACO agreeableness is linked to (a lack of) retaliation in *second-party* punishment^18, 19^, it is unclear whether this would extend to instances of third-party punishment which involve defending the interests of others.

## Results

### Preliminary Statistics

#### Personality variables

Descriptive statistics for prosocial personality traits and their intercorrelations are presented in Table 1. In line with previous research^21, 24^, the politeness aspect of Big Five agreeableness was highly correlated with both HEXACO honesty-humility (*r*_*s*_ = 0.52, *p* < 0.001) and HEXACO agreeableness (*r*_*s*_ = 0.49, *p* < 0.001).

**Table 1:** Descriptive Statistics and Correlations between Personality Variables and Economic Games.

Variable	*N*	Mean (SD)	Correlations					
			1	2	3	4	5	6
(1) B5 Agreeableness	340	3.88 (0.59)	0.91					
(2) B5 Compassion	340	3.84 (0.75)	0.90**	0.93				
(3) B5 Politeness	340	3.91 (0.60)	0.84**	0.54**	0.81			
(4) HEX Honesty-Humility	340	3.43 (0.70)	0.41**	0.26**	0.52**	0.87		
(5) HEX Agreeableness	340	3.14 (0.66)	0.51**	0.40**	0.49**	0.27**	0.89	
(6) Dictator allocation	340	3.41 (2.23)	0.17**	0.13*	0.20**	0.30**	0.01	
***Third-Party Punishment***								
Points spent when A transfers 0	170	1.02 (1.16)	0.09	0.15	0.03	−0.03	−0.001	0.29**
Points spent when A transfers 1	170	0.84 (1.05)	0.07	0.12	−0.004	−0.04	0.03	0.27**
Points spent when A transfers 2	170	0.60 (0.78)	0.10	0.13	0.05	−0.01	0.06	0.30**
Points spent when A transfers 3	170	0.47 (0.68)	0.01	0.07	−0.06	−0.04	0.01	0.28**
Points spent when A transfers 4	170	0.25 (0.50)	0.03	0.08	−0.04	0.01	0.07	0.27**
Points spent when A transfers 5	170	0.13 (0.35)	−0.03	0.03	−0.12	0.0001	0.004	0.19*
Total punishment points	170	3.31 (3.95)	0.07	0.14	−0.01	−0.03	0.01	0.32**
***Third-Party Recompensation***								
Points spent when A transfers 0	170	1.51 (1.44)	0.29**	0.32**	0.19*	0.20**	0.14	0.34**
Points spent when A transfers 1	170	1.32 (1.30)	0.26**	0.28**	0.18*	0.19*	0.12	0.39**
Points spent when A transfers 2	169	1.13 (1.18)	0.19*	0.22**	0.11	0.12	0.13	0.35**
Points spent when A transfers 3	170	1.01 (1.20)	0.16*	0.21**	0.08	0.03	0.12	0.38**
Points spent when A transfers 4	170	0.80 (1.22)	0.11	0.20**	−0.01	−0.01	0.05	0.30**
Points spent when A transfers 5	170	0.68 (1.31)	0.05	0.12	−0.03	−0.06	0.02	0.18*
Total recompensation points	169	6.48 (6.47)	0.22**	0.27**	0.11	0.11	0.11	0.42**

Notes: Correlations calculated using Spearman’s rho. Cronbach’s αs are shown in the diagonal. B5 = Big Five Model, measured using the Big Five Aspect Scales (BFAS)^13^. HEX = HEXACO Model, measured using the HEXACO Personality Inventory—Revised (HEXACO-PI-R)^11^. Dictator game decisions refer to points allocated to a partner out of 10. Third-party punishment (recompensation) decisions refer to points out of 5 spent on deducting (increasing) a dictator’s (recipient’s) payoff.

*p < 0.05. **p < 0.01.

#### Economic games

**Figure 2:**
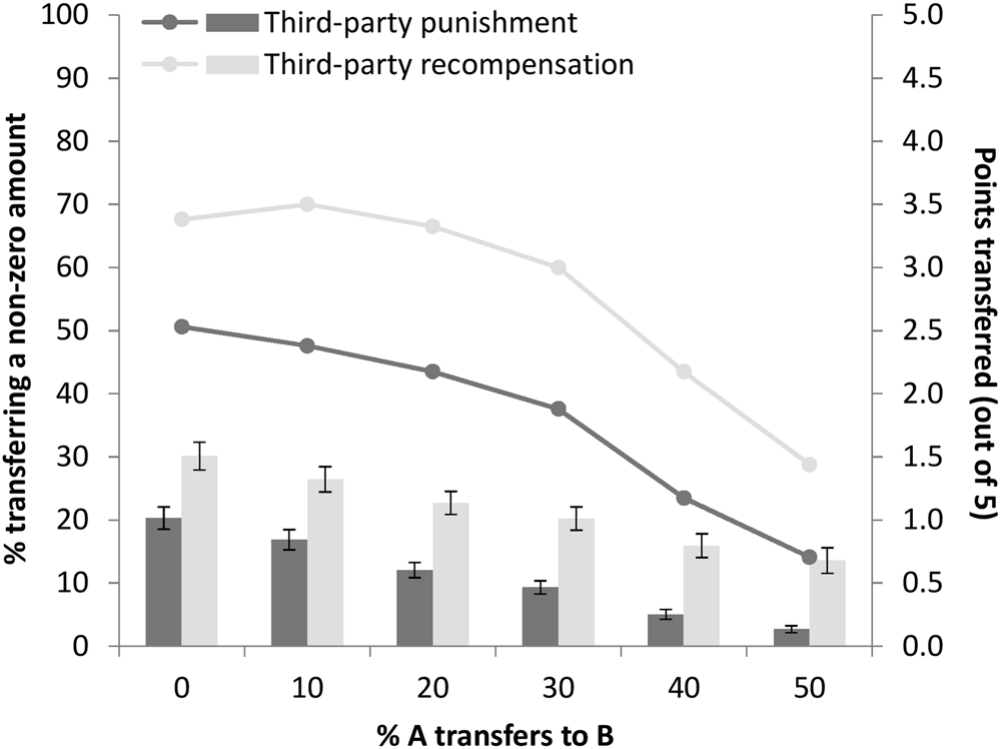
Percentage of sample punishing/recompensing (lines) and average number of points spent on punishment/recompensation (columns) for each level of the dictator transfer in the third-party games. Error bars indicate ± one standard error. *N*s = 170 (punishment), 170 (recompensation).

Descriptive statistics for the dictator, 3PP, and 3PR games are presented in Table 1 and Fig. 2. The average allocation in the dictator game was 34% of the endowment to one’s partner, with 28% of participants choosing to keep the entire amount for themselves. This is similar to typical divisions of wealth reported in a meta-analysis of the dictator game^41^.

In the 3PP game, 51% of participants punished when the dictator transferred nothing to the recipient, with this percentage dropping to 14% when the dictator transferred an equal share to the recipient (McNemar’s test, *p* < 0.001). The average number of points spent on punishment ranged from 1.02 (20% of the participant’s endowment) when the dictator transferred nothing, to 0.13 (2.6% of the endowment) when the dictator transferred equally. The corresponding figures were higher in the 3PR game. Here, the percentage of individuals recompensing was highest (68–70%) when the dictator transferred 0–10% of their endowment and fell to 29% when the dictator transferred equally (McNemar’s test, *p* < 0.001). This corresponded with an average of 1.51 points (30% of the participant’s endowment) spent on recompensation when the dictator transferred nothing and 0.68 (14% of the endowment) when the dictator transferred equally.

#### Prevalence of prosocial and antisocial punishment

Given research highlighting the distinction between prosocial and antisocial punishment^37, 42^, we examined the participants who meted out any punishment in the 3PP game (*N* = 88). Using third-party responses in conjunction with the dictator game, participants were divided into those who were prosocial punishers and antisocial punishers, similar to a previous approach^37^. Seventy-five of these (85%) were prosocial punishers, defined as participants who spent any points on punishment in the 3PP game and who allocated fairly or generously (transfers ≥ 40%) as the dictator in the dictator game. The remaining 13 (15%) were antisocial punishers, defined as participants who spent any points on punishment but who allocated unfairly (transfers ≤ 30%) as the dictator.

### Bivariate Correlations between Prosocial Personality Traits and Game Decisions

Correlations between prosocial personality traits and economic game decisions are presented in Table 1 (see Table S1 in the online supplementary information for correlations with all Big Five traits and aspects). In the dictator game, allocations to a partner were more strongly associated with politeness (*r*_*s*_ = 0.20, *p* < 0.001) than compassion (*r*_*s*_ = 0.13, *p* = 0.02), with the latter no longer significant after controlling for politeness (*pr*_s_ = 0.03, *p* = 0.65). Of the two HEXACO traits, only honesty-humility was correlated with dictator allocations (*r*_*s*_ = 0.30, *p* < 0.001). Dictator allocations were also moderately correlated with punishment (*r*_*s*_s = 0.19–0.30) and recompensation (*r*_*s*_s = 0.18–0.39) for all third-party games.

In the 3PP game, none of the aspects or traits from the Big Five and HEXACO models showed a strong relation with punishment. However, compassion had a marginally significant association with punishment when the dictator transferred nothing to the recipient (*r*_*s*_ = 0.15, *p* = 0.05) and with the total number of points spent on punishment (*r*_*s*_ = 0.14, *p* = 0.07).

In the 3PR game, compassion predicted recompensation in all instances where the dictator did not share equally with the recipient (from *r*_*s*_ = 0.20 when the dictator transferred 40% to *r*_*s*_ = 0.32 when the dictator transferred 0%). When the dictator transferred a minimal share (i.e., 10% or nothing), politeness and honesty-humility also predicted recompensation (*r*_*s*_s = 0.18–0.20, *p*s = 0.01–0.02). Beyond the prosocial traits, there were also significant correlations between total points spent on recompensation and the enthusiasm (*r*_*s*_ = 0.21, *p* = 0.01) and assertiveness (*r*_*s*_ = 0.16, *p* = 0.04) aspects of Big Five extraversion, as well as the openness aspect of Big Five openness/intellect (*r*_*s*_ = 0.15, *p* = 0.05). However, all of the above trait effects disappeared when controlling for compassion (all *p*s > 0.19).

We tested for a double dissociation between prosocial traits and game behaviours in the 3PR game using Steiger’s^43^ method for comparing correlated coefficients. These analyses were conducted using one-tailed probability given the a priori prediction of the double dissociation based on the existing literature. Here, the correlation between dictator allocations and politeness was significantly greater than that with compassion (*r*_*s*_s = 0.27 vs. 0.14), *Z* = 1.88, *p* = 0.03. In addition, the correlation between third-party recompensation and compassion was significantly greater than that with politeness (*r*_*s*_s = 0.27 vs. 0.11), *Z* = 2.23, *p* = 0.01. The same pattern was observed when politeness was replaced with HEXACO honesty-humility. Here, the correlation between dictator allocations and honesty-humility was significantly greater than that with compassion (*r*_*s*_s = 0.30 vs. 0.14), *Z* = 1.75, *p* = 0.04, whereas the correlation between third-party recompensation and compassion was marginally greater than that with honesty-humility (*r*_*s*_s = 0.27 vs. 0.11), *Z* = 1.62, *p* = 0.05.

### Repeated Measures ANCOVA Models of Game Decisions

**Figure 3:**
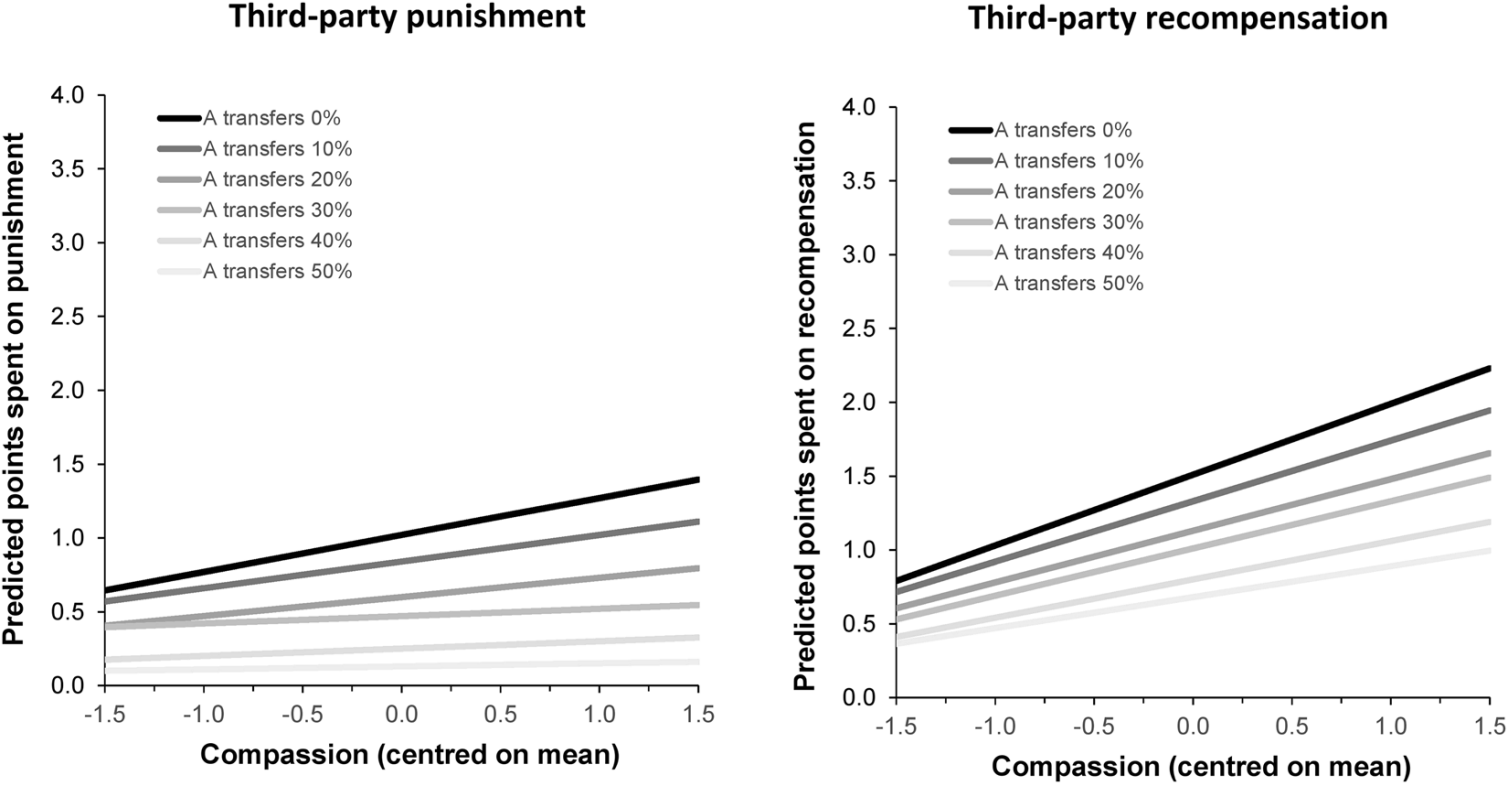
Predicted number of points (out of 5) spent on third-party punishment or recompensation as a function of the compassion aspect of Big Five agreeableness. *N*s = 170 (punishment), 170 (recompensation).

For each third-party game, we performed an analysis of covariance (ANCOVA) with a repeated measures factor of the fairness of the dictator transfer (10:0, 9:1, 8:2, 7:3, 6:4, 5:5) and prosocial traits as continuous covariates. This was performed for the Big Five and HEXAO models separately, with the relevant two prosocial traits standardised and entered simultaneously. We also reran each model with dictator allocations standardised and entered as an additional covariate to control for unconditional giving. Figure 3 presents the separate regression slopes by each level of fairness for the compassion aspect of Big Five agreeableness.

#### 3PP game

In the 3PP game, Greenhouse-Geisser corrections were applied for sphericity violations of fairness, χ^2^(14) = 558.39, *p* < 0.001 (ɛ = 0.37). In the Big Five model, there was a main effect for fairness, *F*(1.85, 308.13) = 76.14, *p* < 0.001, η_p_^2^ = 0.31, and a marginally significant main effect for compassion, *F*(1, 167) = 2.84, *p* = 0.09, η_p_^2^ = 0.02, but not politeness, *F*(1, 167) = 0.41, *p* = *0*.52, η_p_^2^ = 0.002. There were no significant interactions between fairness and each of the aspects of agreeableness (all *p*s > 0.14). When dictator allocations were included in the model, they yielded a main effect, *F*(1, 166) = 18.86, *p* < 0.001, η_p_^2^ = 0.10, and an interaction with fairness, *F*(1.89, 313.85) = 6.85, *p* = 0.002, η_p_^2^ = 0.04. The main effect for compassion disappeared after controlling for dictator allocations, *F*(1, 166) = 1.61, *p* = 0.21, η_p_^2^ = 0.01.

In the HEXACO model, there was a main effect for fairness, *F*(1.83, 305.87) = 75.02, *p* < 0.001, η_p_^2^ = 0.31, but no significant main effects or interactions for each of the HEXACO traits (all *p*s > 0.67). When dictator allocations were included in the model, they produced a main effect and interaction with fairness (as in the Big Five model), but there remained no significant effects for either of the HEXACO traits.

#### 3PR game

In the 3PR game, Greenhouse-Geisser corrections were again applied for sphericity violations of fairness, χ^2^(14) = 788.22, *p* < 0.001 (ɛ = 0.31). In the Big Five model, there was a main effect for fairness, *F*(1.53, 254.53) = 29.15, *p* < 0.001, η_p_^2^ = 0.15, and compassion, *F*(1, 166) = 9.78, *p* = 0.002, η_p_^2^ = 0.06, but not politeness, *F*(1, 166) = 0.43, *p* = 0.51, η_p_^2^ = 0.003. There were no significant interactions between fairness and each of the aspects of agreeableness (all *p*s > 0.68). When dictator allocations were included in the model, they yielded a main effect, *F*(1, 165) = 21.74, *p* < 0.001, η_p_^2^ = 0.12, but no interaction with fairness, *F*(1.53, 252.59) = 0.65, *p* = 0.48, η_p_^2^ = 0.004. While the effect for compassion remained significant, there was also a marginally significant (negative) main effect for politeness after controlling for dictator allocations, *F*(1, 165) = 3.29, *p* = 0.07, η_p_^2^ = 0.02.

In the HEXACO model, there was a main effect for fairness, *F*(1.53, 253.90) = 29.24, *p* < 0.001, η_p_^2^ = 0.15, and a marginally significant effect for HEXACO agreeableness, *F*(1, 166) = 3.51, *p* = 0.06, η_p_^2^ = 0.02, but not honesty-humility, *F*(1, 166) = 0.08, *p* = 0.77, η_p_^2^ = 0.001. When dictator allocations were included in the model, they produced a main effect but no interaction with fairness (as in the Big Five model). While the effect for honesty-humility remained non-significant, there was a significant main effect for HEXACO agreeableness after controlling for dictator allocations, *F*(1, 165) = 5.33, *p* = 0.02, η_p_^2^ = 0.03. However, this effect disappeared when the compassion aspect of Big Five agreeableness was also entered into the same model. Here, only compassion, *F*(1, 164) = 5.09, *p* = 0.03, η_p_^2^ = 0.03, and dictator allocations, *F*(1, 164) = 20.69, *p* < 0.001, η_p_^2^ = 0.11, were unique predictors of recompensation, in addition to fairness.

## Discussion

In this study we examined how individual differences in adherence to fairness norms in the dictator game and third-party interventions to violations of the same norms were related to prosocial personality traits from the Big Five and HEXACO models. The findings showed that dictator allocations and third-party punishment and recompensation were differentially associated with politeness/honesty-humility and compassion. They provide evidence for (at least) two separate mechanisms driving prosociality in economic games, which map onto different domains of personality and may stem from distinct emotional, psychological, biological, and evolutionary antecedents. These findings and their implications will be discussed in the following sections.

Consistent with previous studies, the current research revealed a sizeable proportion of people willing to bear a cost to punish an offender and to assist a victim when fairness norms were violated^30, 36, 44^. Both punishment and recompensation increased with the degree of injustice, although people recompensed more—and spent more money doing so—than they punished. One possibility is that this is an artefact of the constraints of the punishment condition, given that third parties could not deduct more points from the dictator than what they already owned. However, punishment rates and amounts in the current study were generally comparable to those in previous research where losses could be incurred^30^, and average amounts spent on punishment were well below ceiling levels across all conditions. This is an important consideration for how prosociality is measured in economic games, which have disproportionately concentrated on punitive responses to norm violations.

The current findings also allow us to comment on the prosocial nature of third-party punishment. In contrast to previous research and the idea that “punishment is not in general ‘altruistic’” (p. 3)^4^, we showed that dictator allocations were related to both third-party punishment and recompensation (*r*_*s*_s = 0.32, 0.42) and were the strongest predictor of interventions to norm violations over and above those of self-reported traits (which may partly reflect common-method variance). In a similar vein, other studies have shown that forms of active cooperation, such as dictator allocations and cooperation in prisoner’s dilemmas and trust games were positively associated with third-party punishment^45, 46^. Furthermore, there was a relatively small proportion of punishers who behaved antisocially (i.e., those who did not divide the money fairly when they themselves were dictators, *N* = 13 or 15% of all punishers, and those who punished an equitable dictator, N = 24 or 14% of all people in the 3PP game). Previous research suggests that antisocial punishers are motivated by spite and dominance^37, 42, 47^, and the reason for their small numbers may be the absence of situational cues promoting local competition^48^ in the current paradigm.

We further mapped individual differences in these game behaviours to self-reports of personality, which revealed a double dissociation of prosocial traits and behaviours. Specifically, traits reflecting fair-mindedness and non-aggression—politeness and honesty-humility—were associated with more equitable allocations of wealth in the dictator game, consistent with previous research^6, 22–24^, see also^49^. In contrast, compassionate dictators were no more likely than anyone else to share their money after controlling for their good manners.

This pattern of findings was reversed in the third-party games, where participants now witnessed someone else play as the dictator against a powerless recipient. Despite the considerable evidence linking politeness and honesty-humility with acts of active cooperation—dictator allocations, public goods contributions, and returns in the trust game^6, 22–27^—individuals high on these traits were just as likely as anyone else to “turn the other cheek” as a passive bystander, particularly after controlling for their emotional concern. This cannot be explained by a self-interested reluctance to bear a cost for others, as these same individuals were willing to part with their money when they themselves were dictators just moments earlier. Instead, it was the compassionate people who gave up their own money to intervene after witnessing the powerlessness and exploitation of others, which they did largely by recompensing the victim. Together, these findings are consistent with previous research^36, 37, 39^ and fit with the theoretical basis of compassion, which by definition involves sensitivity to the suffering of others and the motivation to alleviate and prevent it^50^.

An alternative but less theoretically-congruent explanation is that compassionate people recompensed out of preferences for efficiency, given that each point spent is tripled on impact. Indeed, efficiency appears to be a concern when the dictator transferred an equal share, with almost 30% of participants recompensing a fairly-treated recipient. Nevertheless, the effect of compassion disappeared in this condition (*r*_*s*_ = 0.12, *p* = 0.13), suggesting that it is specific to the mistreatment of others. This is also in keeping with previous evidence showing no clear positive relations between compassion and socially-maximising acts of resource allocation^51^.

The correlation between compassion and punishment of the offender was somewhat fragile, and at best, reached *r*_*s*_ = 0.15 when the dictator transferred nothing. This relatively weak role of compassion in punishment compared with recompensation may be partly understood through research distinguishing *helping* from *moral courage* as two forms of bystander intervention. While helping (e.g., recompensation) refers to intentional behaviour focused on the needs of a victim, much like compassion^52^, moral courage is defined as intentional norm-enforcing behaviour focused on the perpetrator^53^. Moral courage is accompanied by anger and indignation, and has more in common with dispositional justice sensitivity, self-assurance, and Big Five openness than empathic concern^53, 54^. Other research has found that third-party punishment of cheaters was mitigated when participants were induced to feel compassion towards an unrelated individual, suggesting that the experience of compassion may override punitive sentiments^55^.

In the HEXACO model, the partitioning of prosocial traits according to active (i.e., honesty-humility) and reactive (i.e., agreeableness) cooperation has proved useful in dissociating dictator allocations from second-party punishment^18, 19^. Consistent with its conceptualization as a reactive form of cooperation, HEXACO agreeableness showed a weak link with third-party recompensation that became significant once controlling for dictator allocations. However, this disappeared after controlling for the compassion aspect of Big Five agreeableness, suggesting that it is not so much its core characteristic of forgivingness, but the shared variance reflecting emotional concern for others which drives recompensation. Compassion does not have a direct HEXACO equivalent but appears to migrate between honesty-humility, agreeableness, and emotionality^16^. While the current study focused on the former two, one suggestion for future research is to explore the facet level of the HEXACO model—particularly the *sentimentality* facet of HEXACO emotionality (the tendency to feel strong emotional bonds with others)—in third-party games to see whether this produces a similar effect as compassion.

The differential relations between politeness/honesty-humility and compassion with adherence to fairness norms versus interventions to norm violations demonstrate the multidimensional nature of prosociality. Similar distinctions and potential precursors to these prosocial constructs can be found in the developmental literature, where acts such as sharing (i.e. distributing personal resources fairly) are separate to helping (i.e., meeting another’s instrumental needs) and comforting (i.e., providing support to others in distress)^56–58^. These distinct facets of prosociality depend on different social-cognitive abilities and motivational pathways, and have unique trajectories of development throughout childhood^56–58^. In economic games too, acts of active cooperation and helping may have different emotional antecedents and biological underpinnings, which are reflected in their differential associations with the two aspects of Big Five agreeableness. For example, emotional processes linked to prosocial interventions to norm violations—empathic concern, moral outrage, and anger—are less relevant to politeness than they are to compassion, especially since politeness is characterised by low aggression^59^. Other lines of research have identified oxytocin—which has been putatively linked to trait compassion^59^—as an important neuropeptide modulating interventions to norm violations and the reward of cooperative players in third-party games^60^. These distinctions provide a clue into the evolutionary origins of these prosocial constructs. Politeness and honesty-humility promote compliance with group norms away from cheating and thus may have emerged in cooperative interactions and reciprocal altruism with non-relatives^17, 61^. Compassion promotes affiliation with another individual beyond norm boundaries and may have emerged as an extension of parental nurturance^62, 63^.

Until now, trait effects for compassion in economic games have been elusive^24, 64^ and related constructs have been poorly represented in economic decision-making paradigms. This is because the very nature of most games—decontextualised, rule-based encounters with unidentifiable equals—are set up to inhibit the expression of emotional concern for others. Even third-party recompensation games are a relatively new addition to the traditional repertoire of economic games^44^. This is in stark contrast to the psychological literature, where empathic concern is considered to lie at the core of altruism^52^ and is the mainspring of real-world instances of helping and philanthropy^65, 66^.

To conclude, our findings highlight the multidimensional nature of prosocial behaviours and traits in the context of economic games modelling fairness, punishment, and helping. Politeness and honesty-humility drive fair and cooperative behaviours in the dictator game—traits that contribute to a Good Citizen, but third-party games reveal the importance of compassion and empathic concern for helping and defending others—traits that make a Good Samaritan.

## Methods

### Ethics Statement

This study was approved by the Human Ethics Advisory Group of the Melbourne School of Psychological Sciences, The University of Melbourne, and was conducted in accordance with relevant guidelines and regulations. All participants provided informed consent via an electronic survey according to the established guidelines of the Group.

### Participants

The final sample consisted of 340 United States (US) residents (aged 18–74 years, M_age_ = 35.41, SD = 11.39; 44% female) recruited from the online labour market Amazon Mechanical Turk (MTurk). Details on data exclusions are provided in the Procedure. This sample size was selected to allow over *N* = 136 in each of the two third-party games, which would provide 80% power to detect effect sizes of *r* = 0.21^67^. This was based on previous studies of trait effects in economic games^6, 24^ and the average effect sizes in personality and social psychology overall (i.e., *r*s = 0.21, 0.24)^68, 69^.

Data were collected in two waves (*N*s = 189, 151) as part of a broader research program investigating the role of personality traits in social decision making. Workers were only recruited if they had an approval rating of 98% or higher. They were paid a show-up fee of US$8.00 in the first wave and US$2.00 in the second wave, which differed due to the length of the session. In both waves, participants received an average bonus payment of US$0.50.

### Personality Measures

Participants completed the 100-item Big Five Aspect Scales (BFAS)^13^ which measures five broad trait domains of personality (neuroticism, agreeableness, conscientiousness, extraversion, openness/intellect) and each of their two underlying aspects. The two aspects of agreeableness—politeness and compassion—are each measured using ten items on a 5-point Likert-type scale (1 = strongly disagree, 5 = strongly agree). The BFAS has good test-retest reliability and internal consistency, and has been well validated against other measures of the Big Five^13^.

Participants also completed the revised HEXACO Personality Inventory (HEXACO-PI-R)^11^, which measures six broad dimensions of personality (honesty-humility, emotionality, extraversion, agreeableness, conscientiousness, openness to experience). Each dimension is measured using 16 items on a 5-point Likert-type scale (1 = strongly disagree, 5 = strongly agree). To avoid redundancy following the administration of the BFAS, we only used the 32 items for the prosocial domains, focusing on honesty-humility and agreeableness. Also included were four items from an interstitial facet scale, altruism, representing a blend of HEXACO honesty-humility, agreeableness, and emotionality (e.g., “I have sympathy for people who are less fortunate than I am”). The HEXACO-PI-R has high internal consistency and demonstrates good convergent validity with external variables^11^.

### Economic Games

Participants played one of two variants of a third-party game^30, 36, 37^, depicted in Fig. 1. Both games involved three players and consisted of two stages. In the initial stage of both games, the participant was a passive observer (Person C), who witnessed a dictator transfer between two other players^70, 71^. Here, one player (Person A, or the dictator) was endowed with 10 points and decided to allocate any portion of this to a second player (Person B, or the recipient) in one-point increments up to an equal split (i.e., 10:0, 9:1, 8:2, 7:3, 6:4, 5:5). Dictators could not allocate more than half their endowment to recipients, to allow comparability with previous versions of third-party games^30, 36^ and to account for the fact that such “hyper-fair” allocations are rare in standard dictator games^41^.

The two games diverged from one another in the second stage. In the third-party punishment (3PP) game (*N* = 170), the participant was endowed with 5 points and could spend any portion of this to deduct points from the dictator by a factor of three. Therefore, for each punishment point spent, the dictator’s total payoff was reduced by three points. Note, however, that participants could not punish the dictator more than what the dictator already owned. In the third-party recompensation (3PR) game (*N* = 170), the participant was endowed with 5 points and could spend any portion of this to increase the recipient’s points by a factor of three. Therefore, for each recompensation point spent, the recipient’s total payoff increased by three additional points. Decisions were measured using the strategy method^72^. Specifically, participants indicated how much they would punish or recompense for each of the six possible transfers that the dictator made, which ranged in fairness from most unfair (10:0) to fair (5:5).

All participants also played in the role of dictator in a separate dictator game with a new partner^70, 71^. They were asked to indicate their preferred choice out of 11 different payoff combinations between themselves and a recipient, all of which summed to 10 points and varied by one-point increments (i.e., 10:0, 9:1, 8:2, 7:3, 6:4, 5:5, 4:6, 3:7, 2:8, 1:9, 0:10). To retain the basic structure and choice set of the standard game, dictators could allocate more than half their endowment to the recipient, although only one participant did this. Unlike the third-party games, there were no opportunities for other players to respond to this decision.

In all games, participants were informed that their partners were anonymous strangers that they would not knowingly meet. Terms such as “player”, “game”, “punishment”, and “recompensation” were not used to avoid priming participants with connotations of competition.

### Procedure

All demographic questions, personality measures, and economic games were programmed using Qualtrics Survey Software and administered through the MTurk requester interface. In both waves of data collection, this survey included additional questionnaires and decision making tasks beyond the scope of the current study (see Table S2 in the online supplementary information).

Participants first completed the dictator game and were then randomly assigned to either the 3PP or 3PR game. All game decisions were incentivised by informing participants that their decisions in one of the games (which was pre-selected) would be matched to those of other participants and used to determine their payment at the end of the session. All points were then converted to real money at the rate of 1 point = USD$0.10. This approach is based on the Conditional Information Lottery^73^, a standard procedure in studies of economic games which allows researchers to measure a range of game behaviours without deception.

At the conclusion of the third-party game, participants completed three comprehension checks asking them to calculate the final payoffs of Persons A, B, and C for a given set of decisions by Persons A and C. They were deemed to have passed the comprehension checks when the sum of final payoffs was within one point of the correct answer. Deviations of one appeared to be simple arithmetic errors and were not consistent with a fundamental misunderstanding of the game. Based on this, 55 and 52 participants (24% and 23%) were excluded from the final samples of the 3PP and 3PR games, respectively. These proportions are relatively high, but are comparable or lower than those of comprehension difficulties in other economic games^40^. Importantly, all main findings were replicated when the analysis was re-run with the full sample of participants (*N*s = 225, 222 in the 3PP and 3PR games, respectively). That is, in the Big Five model, adherence to fairness norms in the dictator game was uniquely associated with only politeness, while recompensation in the 3PR game was uniquely associated with only compassion. Some of the weaker findings also reached significance in the full sample, including the positive relation between compassion and punishment in the 3PP game.

Following the economic games, participants completed the BFAS and later, the honesty-humility, agreeableness, and altruism scales of the HEXACO-PI-R. Two attention checks were embedded within these questionnaires (e.g., “Please select Strongly Agree”). Thirty-one participants were excluded from the final sample for failing at least one of these checks. The median time spent on the study was 42.18 minutes in the first wave and 24.05 minutes in the second wave of data collection.

## Electronic supplementary material

supplementary information

Supplementary Dataset 1
