# Supplementary material for: Politeness and Compassion Differentially Predict Adherence to Fairness Norms and Interventions to Norm Violations in Economic Games
Source: Sci Rep. 2017 Jun 13;7:3415. doi: 10.1038/s41598-017-02952-1 (PMC5469794; doi:10.1038/s41598-017-02952-1)
Supplement: Supplementary file 1 — supplementary information [file 41598_2017_2952_MOESM1_ESM.pdf]

Politeness and Compassion Differentially Predict Adherence to Fairness Norms and Interventions to Norm Violations in Economic Games

Online Supplementary Information

Kun Zhao*
The University of Melbourne, Australia

Eamonn Ferguson
University of Nottingham, United Kingdom

Luke D. Smillie
The University of Melbourne, Australia

Author Note
*Correspondence and requests for materials should be addressed to Kun Zhao, Melbourne School of Psychological Sciences, Redmond Barry Building, The University of Melbourne, Victoria, 3010, Australia.
E-mail: kun.zhao@unimelb.edu.au

Table S1

Descriptive Statistics and Correlations between All Personality Traits and Economic Games

Variable	Mean (SD) (*N* = 340)	Correlations: Dictator game (*N* = 340)	Third-party punishment game (*N* = 170)	Third-party recompensation game (*N* = 169)
Big Five Model				
Neuroticism	2.61 (0.85)	.13*	.05	.03
Withdrawal	2.75 (0.93)	.14**	.03	.04
Volatility	2.46 (0.89)	.09	.06	.003
Agreeableness	3.88 (0.59)	.17**	.07	.22**
Compassion	3.84 (0.75)	.13*	.14	.27**
Politeness	3.91 (0.60)	.20**	-.01	.11
Conscientiousness	3.57 (0.63)	.03	-.05	-.02
Industriousness	3.59 (0.77)	-.02	-.07	.01
Orderliness	3.55 (0.66)	.08	-.05	-.05
Extraversion	3.18 (0.76)	-.07	.06	.22**
Enthusiasm	3.25 (0.83)	-.02	.06	.21**
Assertiveness	3.11 (0.87)	-.10	.03	.16*
Openness/Intellect	3.82 (0.62)	-.04	.02	.16*
Openness	3.81 (0.68)	.01	.11	.15*
Intellect	3.84 (0.73)	-.08	-.09	.12
HEXACO Model				
Honesty-Humility	3.43 (0.70)	.30**	-.03	.11
Agreeableness	3.14 (0.66)	.01	.01	.11
Altruism	3.92 (0.72)	.16**	.12	.10

Notes: Correlations calculated using Spearman’s rho. B5 = Big Five Model, measured using the Big Five Aspect Scales (BFAS)[1]. HEX = HEXACO Model, measured using the HEXACO Personality Inventory—Revised (HEXACO-PI-R)[2]. Dictator game decisions refer to points allocated to a partner out of 10. Third-party punishment (recompensation) decisions refer to points out of 5 spent on deducting (increasing) a dictator’s (recipient’s) payoff.
*p < .05. **p < .01.

Table S2

Additional Tasks and Questionnaires Administered in the Current Study

Measure	Reference	Wave Administered	
		Wave 1	Wave 2
Tasks			
Giving- or taking-framed dictator game	Adapted for this study	X	
Moral dilemma	Adapted for this study	X	
Social mindfulness task	Van Doesum, N. J., Van Lange, D. A.W., & Van Lange, P. A. M. (2013). Social mindfulness: Skill and will to navigate the social world. *Journal of Personality and Social Psychology*, *105*(1), 86–103.	X	
Conflict templates	Halevy, N., Chou, E. Y., & Murnighan, J. K. (2012). Mind games: The mental representation of conflict. *Journal of Personality and Social Psychology*, *102*(1), 132–148.	X	
Public goods game	Hilbig, B. E., Zettler, I., & Heydasch, T. (2012). Personality, punishment and public goods: Strategic shifts towards cooperation as a matter of dispositional Honesty-Humility. *European Journal of Personality*, *26*, 245–254.		X
Questionnaires			
Interpersonal Reactivity Index	Davis, M. H. (1980). A multidimensional approach to individual differences in empathy. *JSAS Catalog of Selected Documents in Psychology*, *10*, 85.	X	
Major Life Goals	Roberts, B. W., & Robins, R. W. (2000). Broad dispositions, broad aspirations: The intersection of personality traits and major life goals. *Personality and Social Psychology Bulletin, 26*(10), 1284–1296.	X	
Propensity to Trust Survey	Evans, A. M., & Revelle, W. (2008). Survey and behavioral measurements of interpersonal trust. *Journal of Research in Personality, 42*(6), 1585–1593.	X	
Altruistic Personality Scale	Rushton, J. P., Chrisjohn, R.D., & Fekken, G. C. (1981). The altruistic personality and the self-report altruism scale. *Personality and Individual Differences, 1*, 292-302.	X	
Dirty Dozen measure of the Dark Triad	Jonason, P. K., & Webster, G. D. (2010). The Dirty Dozen: A concise measure of the Dark Triad. *Psychological Assessment, 22*(2), 420.	X	
Big Five Inventory-2	Soto, C. J., & John, O. P. (2016). The next Big Five Inventory (BFI-2): Developing and assessing a hierarchical model with 15 facets to enhance bandwidth, fidelity, and predictive power. *Journal of Personality and Social Psychology*. Advance online publication.		X

References

1. DeYoung, C. G., Quilty, L. C. & Peterson, J. B. Between facets and domains: 10 aspects of the Big Five. *J. Pers. Soc. Psychol.* **93,** 880–896 (2007).

2. Lee, K. & Ashton, M. C. Psychometric properties of the HEXACO Personality Inventory. *Multivariate Behav. Res.* **39,** 329–358 (2004).
